# Relationship between Branched-Chain Amino Acids, Metabolic Syndrome, and Cardiovascular Risk Profile in a Chinese Population: A Cross-Sectional Study

**DOI:** 10.1155/2016/8173905

**Published:** 2016-07-26

**Authors:** Wen Hu, Luning Sun, Yingyun Gong, Ying Zhou, Panpan Yang, Zhengqin Ye, Jinxiang Fu, Aijie Huang, Zhenzhen Fu, Weinan Yu, Yang Zhao, Tao Yang, Hongwen Zhou

**Affiliations:** ^1^Department of Endocrinology, The First Affiliated Hospital of Nanjing Medical University, Nanjing 210029, China; ^2^Department of Endocrinology and Metabolism, Huai'an Hospital Affiliated to Xuzhou Medical University and Huai'an Second People's Hospital, Huai'an 223001, China; ^3^Research Division of Clinical Pharmacology, The First Affiliated Hospital of Nanjing Medical University, Nanjing 210029, China; ^4^School of Public Health, Nanjing Medical University, Nanjing 210029, China

## Abstract

*Objective.* This study aimed to evaluate the relationship between branched-chain amino acids (BCAAs), metabolic syndrome (MS), and other cardiovascular (CV) risk factors in middle-aged and elderly Chinese population at high risk for the development of cardiovascular disease (CVD).* Methods.* 1302 subjects were enrolled from the Huai'an Diabetes Prevention Program.* Results.* BCAAs levels were positively correlated with MS, its components, and CV risk profile. The odds ratio (OR) for MS among subjects in the fourth quartile of BCAAs levels showed a 2.17-fold increase compared with those in the first quartile. BCAAs were independently associated with high Framingham risk score even after adjusting for MS and its components (*P* < 0.0001). Additionally, the OR for high CV risk was 3.20-fold (*P* < 0.0001) in participants in the fourth BCAAs quartile with MS compared with participants in the first BCAAs quartile without MS.* Conclusions.* Increased BCAAs levels are independent risk factors of MS and CVD in addition to the traditional factors in middle-aged and elderly Chinese population. The development of CVD in MS patients with high level BCAAs is accelerated. Intervention studies are needed to investigate whether the strategy of BCAAs reduction has impacts on endpoints in patients with higher CV risk. This study is registered with ChiCTR-TRC-14005029.

## 1. Introduction

Cardiovascular disease (CVD) is the major cause of death in developed countries and some developing countries [1]. Though conventional risk prediction algorithms are made available on presence of major cardiovascular (CV) risk factors, further investigations on authentic and accurate biomarkers of CVD are needed. To date, numerous physiological biomarkers based on serum lipids, glucose, and hormones associated with CV risks have been identified [2]. Moreover, metabolomics has also been applied to human disease studies. In 2010 Shah et al. performed quantitative mass spectrometry-based metabolic profiling in 117 individuals within eight multiplex families from the GENECARD study of premature coronary artery disease (CAD), and results revealed that BCAAs and its metabolites were highly heritable and could distinguish families with premature coronary artery disease [3]. Therefore, CV risks are not only restricted to carbohydrates and fatty acid metabolism disorders, but also associated with altered protein and amino acid metabolism disorders [3, 4].

The branched-chain amino acids (BCAAs) (leucine (Leu), isoleucine (Ile), and valine (Val)) are essential amino acids that cannot be synthesized de novo [5]. However, excessive intake of amino acids or inborn errors in the genes encoding for the catalytic enzymes in the BCAA catabolism pathway cause accumulation of BCAAs and its metabolites [6], which may produce pathological changes ranging from neurological distress to cardiomyopathy [7]. Maple syrup urine disease (MSUD) is a Mendelian disease due to deficiency of the branched-chain ketoacid dehydrogenase complex (BCKDC) and associated with elevations in the BCAAs and their ketoacids [8]. Some studies [7, 9] on MSUD also demonstrated that missing a key regulator in the catabolism of BCAAs could cause a significant impairment in cardiac function via mechanism of elevated reactive oxygen species (ROS) levels and hypersensitive calcium-induced permeability transition pore opening in the mitochondria of the myocardium. Moreover, a recent case-control study based on circulation metabolic profile of peripheral blood has demonstrated a link between abnormal metabolism of BCAAs and coronary diseases [3]. Simple metabolite profiles are independently associated with CAD and the occurrence of subsequent CV events. These profiles point toward potential diverse and novel mechanisms of CAD pathophysiology and the opportunity for improved risk stratification. It is indicated that the high BCAAs levels may have an impact on cardiac pathology in both hereditary disease and common chronic disease [3, 8]. However, it is not clear whether BCAAs can be applied for identifying subjects with CVD in general population, particularly subjects with metabolic syndrome (MS) at high risk for the development of CVD.

MS is a cluster of abnormalities, such as hypertension, dyslipidemia, abdominal obesity, and insulin resistance (IR). MS is traditional risk factor associated with CV disease, stroke, and all-cause mortality in the general population [10]. A recent Mexico study evaluated the association between MS, homeostasis model assessment of insulin resistance (HOMA-IR), and BCAA levels in 115 Mexican subjects with different degree of obesity; the results showed that subjects with MS had a serum BCAA concentration approximately 34% higher than that in subjects without MS, suggesting that BCAAs levels were possibly associated with MS [11]. Some reports described an association between BCAAs, IR, and obesity [12], indicating that BCAAs concentration is a predictor of the progression of diseases such as diabetes [13]. Therefore, the BCAAs levels may play an important role in predicting relevant components of the MS which are independently risk factors for CVD. However, the relationship between BCAAs, MS, and CVD has not been clearly elucidated, especially in Asian populations.

This study raises the question about whether serum BCAAs levels and MS are merely different aspects of the same risk phenotype or whether they actually perform independent clinical prognostic value for CVD. Unraveling the complex interaction between BCAAs, MS, and other CV risk factors might be helpful for improving preventive and therapeutic strategies for CVD. For this aim, the association of increased BCAA levels with MS and CV risk profile was evaluated in a Chinese population.

## 2. Materials and Methods

### 2.1. Ethics Statement

This cross-sectional study was part of the Huai'an Diabetes Prevention Program (HADPP, ChiCTR-TRC-14005029) with residents attending a physical examination and approved by the Huai'an Second Hospital Ethics Committee of the Xuzhou Medical University School of Medicine (Huai'an city, Jiangsu Province, China). Written informed consent was obtained from all of the participants in this study.

### 2.2. Study Population

**Figure 1:**
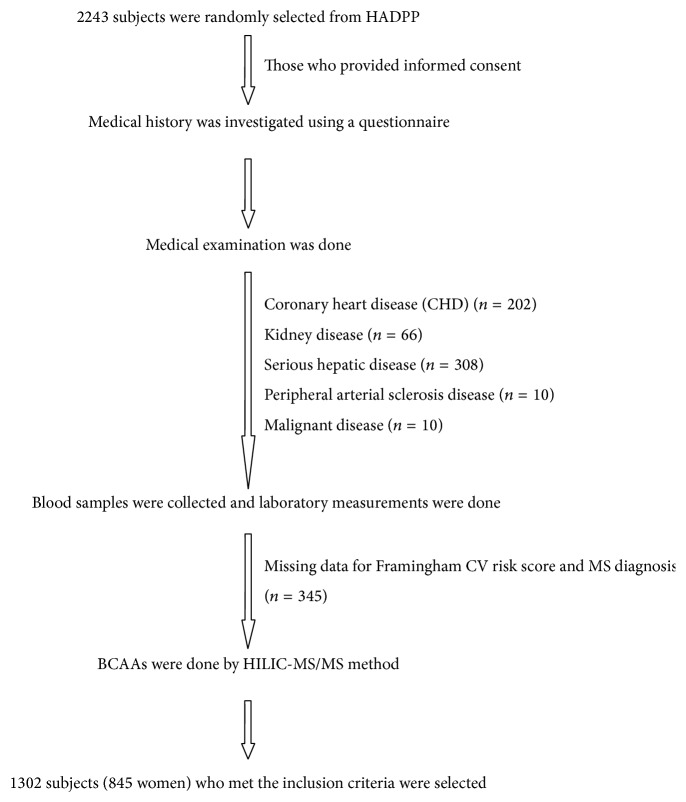
Subjects selection process.

This study recruited 2243 participants, ranging from 40 to 79 years of age, in the framework of routine health examinations in Huai'an city in Jiangsu Province from August to September 2014. Registered criteria were as follows: (1) age <80 years; (2) being healthy by self-evaluation (without headaches, syncope, chronic cough, wheezing, dyspnea, palpitations, and chest pain); (3) the ability to perform self-care and instrumental activities of daily living without difficulties or need for help; and (4) the ability to provide self-reported data and informed consent. Subjects' selection process was shown in Figure 1. We excluded participants with (1) coronary heart disease, including myocardial infarction and angina pectoris (*n* = 202); (2) previously diagnosed kidney disease, including nephritis, autoimmune or drug-induced kidney disease, renal failure, or kidney transplant with dialysis treatment (*n* = 66); (3) previously diagnosed serious hepatic disease, including fatty liver, liver cirrhosis, and autoimmune hepatitis (*n* = 308); (4) peripheral arterial sclerosis disease (*n* = 10); (5) any malignant disease (*n* = 10); or (6) missing data for Framingham CV risk score and MS diagnosis (*n* = 345). Finally, a total of 1302 participants (845 women) were eligible for the analysis.

### 2.3. Data Collection

The demographic characteristics, lifestyle information, and medical history were obtained by trained investigators through a standard questionnaire. The body mass index (BMI) was calculated as the subject's weight (kg) divided by their height squared (m^2^). Waist circumference (WC) was measured with a nonstretchable tape over the unclothed abdomen at the narrowest point between the lowest rib and the iliac crest. Two measures were made and the mean (expressed in centimetres) was used for analyses. The subject's blood pressure (BP) was consecutively measured for three times (Omron Model HEM-752 FUZZY, Omron Company, Dalian city, Liaoning Province, China), and the mean reading was used for the analysis. After an overnight fasting, venous blood samples were collected between 07:00 and 09:00 for measurement of the fasting blood glucose (FPG). The serum creatinine; uric acid (UA); lipid profiles (total cholesterol (TC), triglycerides (TG), low density lipoprotein cholesterol (LDL-C), and high density lipoprotein cholesterol (HDL-C)); and haemoglobin A1c (HbA1c) levels were measured using high-performance liquid chromatography (VARIANT II and D-10 Systems, Bio-Rad, Hercules, CA, USA).

The estimated glomerular filtration rate (eGFR) was calculated from the creatinine levels using the Chronic Kidney Disease Epidemiology Collaboration (CKD-EPI) formula [14].

BCAAs (L-Leu, L-Ile, and L-Val) were detected by a simple, robust, and sensitive hydrophilic interaction chromatography-tandem mass spectrometric (HILIC-MS/MS) method using stable isotope-labeled amino acids as internal standard [15]. Chromatographic separation was carried out on a Syncronis HILIC column (150 mm × 2.1 mm, 5 *μ*m) with the column temperature of 35°C and a mobile phase consisted of 120 mmol/L ammonium acetate buffer-acetonitrile containing 20 mmol/L ammonium acetate (89 : 11, v/v). The retention times for L-Val, L-Leu, and L-Ile were 8.45 min, 5.33 min, and 5.96 min, respectively. The mass spectrometric analysis was performed using a QTrap 5500 mass spectrometer coupled with an electrospray ionization (ESI) source on positive ion mode. The multiple reaction monitoring (MRM) transitions of* m/z *118.0 → 72.1, 132.1 → 86.1, and 132.1 → 86.1 were used to quantify those three amino acids. The MRM transitions of* m/z *126.0 → 80.1, 142.0 → 96.1, and 138.1 → 91.1 were used to quantify corresponding stable isotope-labeled amino acids. The matrix effect was 98.7~107.3%; the recovery was 92.7~102.3%. Analytes were stable during the study. Calibration curve using water instead of serum was linear over the range of 0.2–100 *μ*g/mL. The lower limit of quantification was 0.2 *μ*g/mL. The accuracy and inter- and intraprecision using water instead of serum were below 10.2%.

### 2.4. Definitions

The presence of MS was defined according to the Adult Treatment Panel III (ATP III) criteria as the presence of three or more of the following risk factors [16]: (1) obesity: a WC >90 cm in men and >80 cm in women; (2) elevated TG: a serum TG level >1.70 mmol/L (150 mg/dL); (3) reduced HDL-C: an HDL-C level <1.04 mmol/L (40 mg/dL) in men or <1.30 mmol/L (50 mg/dL) in women; (4) elevated BP: BP >130/85 mmHg and/or the use of antihypertensive medications; and (5) elevated FPG: a serum glucose level >6.11 mmol/L (110 mg/dL) and/or the use of insulin or hypoglycemic medication.

The presence of diabetes was defined according to the 2012 American Diabetes Association (ADA) criteria as follows [17]: FPG ≥126 mg/dL (7.0 mmol/L) or 2 h plasma glucose in the 75 g OGTT ≥200 mg/dL (11.1 mmol/L) or HbA1c ≥6.5%.

Framingham risk score was calculated in each patient based on the risk charts published by D'Agostino Sr. et al. [18] for the prediction of 10-year fatal and nonfatal CV events. Both current and former smokers were included in the category of smokers. High Framingham CV risk was defined as ≥20%.

### 2.5. Statistical Analysis

The continuous variables are presented as means ± standard deviations (SDs) or median with range (minimum, maximum); categorical variables are presented as numbers (%). For analysis, the subjects were divided into four groups based on stratification of BCAAs levels using the 25th, 50th, and 75th percentiles as cut-off points. For BCAAs, the groups were as follows: I, BCAAs < 62.06 *μ*g/mL; II, BCAAs 62.06–70.01 *μ*g/mL; III, BCAAs 70.02–79.57 *μ*g/mL; and IV, BCAAs > 79.57 *μ*g/mL. Comparisons between groups were made using means and proportions were compared by ANOVA for continuous data and the chi-square test or Fisher's exact test for categorical data. Data that were not normally distributed were Napierian logarithmically transformed before analysis. Spearman correlation coefficients (*r*-values) were calculated to determine the relationship between metabolic parameters and amino acids.

Multiple logistic regression analysis was used to describe the relationship between BCAAs (Leu, Ile, and Val) and MS. Two models were constructed for each component: the first model was adjusted for age and gender; the second model was adjusted for age, gender, current smoking status and drinking, and the administration of angiotensin converting enzyme inhibitors (ACEI) or angiotensin receptor blockers (ARBs) and MS components (SBP, DBP, WC, TG and HDL-C, and FPG). Multiple logistic regression analyses of the relationship between BCAAs and high Framingham CV risk were used. Three models were constructed for each component: the first model was not adjusted; the second model was adjusted for MS and eGFR; and the third model was adjusted for traditional cardiometabolic risk factors (SBP, DBP, FPG, TG, HDL-C, UA, BMI, eGFR, current drinking and smoking status, and administration of ACEI or ARBs). Moreover, we performed multiple logistic regression analyses of the relationship between BCAAs quartiles, MS, and high Framingham CV risk. Three models were constructed for each component: the first model was not adjusted; the second model was adjusted by eGFR; and the third model was adjusted for traditional cardiometabolic risk factors. *P* values for the trends were calculated by Spearman correlation analysis of categorical variables and odds ratios (ORs) for the different groups, respectively. *P* < 0.05 was considered statistically significant. All statistical analyses were performed using SPSS 16.0 (SPSS Inc., Chicago, IL, USA).

## 3. Results

### 3.1. Baseline Characteristics of the Study Population

A total of 1,302 participants aged from 40 to 79 years old were enrolled (including 845 females and 457 males) and divided into four groups according to the BCAAs quartiles. As shown in Table 1, most of the characteristics evaluated differed among the four groups, except for systolic and diastolic BP, administration of ACEI or ARBs, TC, eGFR, and LDL-C. As BCAAs quartiles increased, subjects were more likely to be older and had higher prevalence of MS and high CV risk. Moreover, there was a significant trend toward higher BMI, WC, FPG, serum creatinine, the ratio of male to female, the prevalence of hypertension, and more unfavorable lipid profile.

Even adjusting for age and gender, the cardiometabolic risk factors (BMI, WC, FPG, HDL-C, and TG), the number of MS components, and the high Framingham CV risk score were different according to BCAAs quartiles.

### 3.2. Spearman Correlation Analysis Highlights Positive Correlation between BCAAs and Traditional Cardiometabolic Risk Factors

The data showed the correlation between total BCAAs as well as individual BCAAs (Leu, Ile, and Val) and traditional cardiometabolic risk factors (Table 2). The total BCAAs as well as individual BCAAs were positively associated with several cardiometabolic risk factors, such as WC, BMI, FPG, HbA1c, TG, UA, and the number of MS components, whereas they were negatively correlated with HDL-C, but not correlated with systolic BP (SBP), diastolic (DBP), TC, or eGFR. Moreover, there were positive correlations between total BCAAs as well as individual BCAAs and high Framingham CV risk score.

### 3.3. Multiple Logistic Regression Analysis Indicating BCAAs as Predictive Factors for MS

The unadjusted and multivariable-adjusted ORs for the association of BCAAs with MS are reported in Table 3. After adjusting for age, gender, current smoking status and drinking, and the administration of ACEI or ARB and MS components (SBP, DBP, WC, and TG and HDL-C; FPG), the OR for MS among subjects in the fourth BCAAs quartile showed a 2.17-fold increase compared with those in the first quartile. Furthermore, the association between the BCAAs and MS was analyzed in two repeated models, respectively. The data showed an independent relationship between individual BCAAs (L-Leu, L-Ile, and L-Val) and MS.

### 3.4. Multiple Logistic Regression Analysis for High Framingham CV Risk Indicating Increased CV Risk due to the Interplay of BCAAs and MS

As shown in Table 4, we analyzed the association between the BCAAs and high Framingham CV risk. After adjusting for MS and eGFR, OR (95% confidence intervals (CIs)) for Framingham high risk class for each 1 *μ*g/mL increase in Leu, Ile and Val were 1.06 (1.02~1.10, *P* = 0.001), 1.17 (1.11~1.22, *P* = 0.001), and 1.05 (1.03~1.07, *P* < 0.001), respectively. After adjusting for SBP, DBP, FPG, TG, HDL-C, UA, BMI, eGFR, alcohol abuse, and administration of ACEI or ARBs, BCAAs levels were independently associated with high Framingham CV risk.

To better clarify the relationship among BCAAs levels, MS, and CV risk status, subjects were divided into eight subgroups on the basis of BCAAs quartiles and the presence/absence of MS (Table 5). The risk for high Framingham CV risk score was stepwise increased as BCAAs levels increased whether in the presence of MS or not. After adjusting for traditional cardiometabolic risk factors, the OR for high Framingham CV risk was 3.20-fold (95% CI 1.83–5.61; *P* < 0.0001) in participants in the fourth BCAAs quartile with MS compared with participants in the first BCAAs quartile without MS.

## 4. Discussion

The association between BCAAs, MS, and CVD in Asian population remains unclear. Our data provide novel and intriguing insights into the complex relationship between BCAAs, MS, and other CV risk profiles in a middle-aged and elderly Chinese population free of CVD. Firstly, BCAAs were positively correlated with many MS components, such as elevated TG, elevated WC, and decreased HDL-C. There was almost 50% higher risk for MS in the fourth BCAAs quartile compared to the first quartile even after adjusting for each MS component. Secondly, after adjusting for traditional CV risk factors, total BCAAs and individual BCAAs (L-Leu, L-Ile, and L-Val) were independently associated with high Framingham CV risk score in the present study. Finally, interaction between BCAAs and MS increased CVD risk after adjusting for traditional CV risk factors.

Our study differs from previous investigations. Firstly, subjects from this cross-sectional study were Chinese district population free of CAD, peripheral arterial disease, and chronic kidney disease (CKD), and medical examinations are applied every two years. Secondly, a novel risk pattern comprising BCAAs, MS, and extensive traditional CV risk factors, including age, gender, BMI, cigarette smoking, alcohol abuse, SBP, DBP, TG, TC, HDL-C, LDL-C, FBG, UA, and eGFR, was applied. Thirdly, the study recruited 1302 individuals. To our knowledge, the present work firstly assesses the associations between BCAAs, MS, and CV risk in large Chinese population.

BCAAs (Leu, Ile, and Val) are human body essential amino acids which are not only raw materials of protein synthesis but also regulators of protein synthesis [19]. BCAAs are important energy materials which produce adenosine triphosphate by oxidative decarboxylation and regulate cell growth, autophagy, neurotransmitter synthesis, carbohydrate utilization, and lipid metabolism [20, 21]. However, the idea that BCAAs or their supplementation might have a positive role in preventing metabolic disease is controversial. It is thought that excess amino acids intake and impairments in BCAAs metabolism could result in accumulation of serum BCAAs in animal and clinical investigations [22, 23]. Therefore, BCAAs are usually acknowledged evidence of metabolism dysfunction. Frequently, recent studies show that BCAAs have been associated with IR, obesity, and diabetes. In a study by Newgard [24], more than 100 analytes were measured in plasma samples from obese and insulin-resistant versus lean and insulin-sensitive subjects. Surprisingly, the components most strongly associated with insulin sensitivity were not lipid-related, but rather comprised of BCAAs (Val, Leu, and Ile) and aromatic amino acids (AAAs) (phenylalanine (Phe) and tyrosine (Tyr)). The preferential association of this BCAA-related metabolite cluster as a new risk factor with IR, type 2 diabetes, and CVD was discussed in many cross-sectional studies [3, 25, 26]. These studies demonstrate the strong and prior association of the BCAA-related metabolite cluster and IR in studies of different design (case-control or cross-sectional) and across multiple ethnic groups and geographical locales.

Few studies discussed the relationship between BCAAs and MS. In our study the percentage of individuals with 3~5 MS components stepwise increased as BCAAs levels elevated; and the percentage of participants with five MS components was highest among participants in the fourth BCAAs quartile (6%). Furthermore, after adjusting for several potential confounders, Leu, Ile, and Val were positively and independently associated with MS. Therefore, higher BCAAs levels could be regarded as significant predictors of MS. However, the mechanism is unclear. On the one hand, several recent studies indicate that BCAAs could induce IR [27]. BCAAs, especially L-Leu, are highly effective activators of mTOR signaling pathway; and persistent activation of mTORC1 promotes IR through serine phosphorylation of insulin receptor substrate- (IRS-) 1 and IRS-2 [12, 24, 28]. On the other hand, the presence of IR and/or MS contributed to increased BCAAs levels due to downregulated expression of the two key BCAA catabolic enzymes (branched-chain-amino-acid aminotransferase 2 and branched-chain alpha-keto acid dehydrogenase complex E1a) in adipose tissue [29]. These findings suggest the complex relationship among BCAAs levels, MS, and IR status. Accordingly, in the present study, it was inferred that the increased BCAAs levels as a new factor for CVD could be a component of MS.

Some studies showed independent association between BCAAs metabolism and CVD [3, 30]. In 2010, Shah and colleagues [3] concluded that BCAAs were positively and independently associated with the presence of CAD. However, they subsequently found that BCAAs were negatively associated with all-cause death and all-cause death plus myocardial infarction in a cohort study with a median follow-up of 3.1 years [30]. The reason of these seemingly discrepant results may be related to the fact that no adjustment for dyslipidemia was made in the multivariable clinical model in the second study. Moreover, the above two studies focused on the role of BCAAs in all patients undergoing cardiac catheterization and the higher CAD prevalence (60% with 1~3 diseased vessels). In our study we recruited 1302 subjects free of CAD, CKD, and peripheral arterial disease and confirmed that BCAAs levels were independently associated with high Framingham CV risk score adjusted by traditional CV risk factors. The underlying mechanisms for the adverse effects of BCAA catabolic defects on the heart remain to be established. Studies show that BCAAs are not only important nutrient source, but also potent signaling molecules which are highly effective activators of mTOR signaling pathway [31]. In turn, increased mTOR activity promotes cardiac hypertrophy, suppresses cardioprotective autophagy, and impairs bioenergetic regulation [32].

Few investigations about relationships between BCAAs and CVD were performed in Asian population. A Chinese study [33] recruited 472 subjects and evaluated relationships between BCAAs and carotid intima-media thickness (cIMT), which is a risk factor of CAD. In agreement with our study, they concluded that BCAAs are independently correlated with increased cIMT. However, a large-scale, cross-sectional, and prospective analysis [26] of ethnicity, amino acids, and diabetes in a South Asian and European cohort reported that the increased levels of Ile, Tyr, alanine, and glutamine were seen in more centrally obese South Asian men. They explained that altered amino acids metabolism in the liver, kidneys, muscle, or adipose tissue is due to different races. South Asian individuals have lower muscle mass and more hepatic fat than Europeans. Therefore, there were some difference in amino acids and CAD in an Asian population compared to Western population. Interestingly, in our study, AAAs (L-Phe, L-Tye) were not positively correlated with CV risk (data not shown). Accordingly, a large multicenter study is necessary to evaluate the relationship between the serum amino acids and CVD in Asian population.

Another main finding of our present report is the detailed characterization of the relationship between BCAAs, MS, and CVD risk. In our study, the increased BCAAs levels showed a significant, additive role for the presence of MS in identifying patients with the higher Framingham CV risk score. Interestingly, compared with the participants in the first BCAAs quartile without MS, BCAAs in the participants in the first BCAAs quartile with MS did not show any association with high Framingham CV risk even after adjusting for several potential confounders. However, in the fourth BCAAs quartile without or with MS, approximately 2.6-fold and 3.2-fold increases in the risk for high Framingham CV risk score were observed, suggesting the interplay between BCAAs and MS contributing to the increased CV risk.

There are several probable mechanisms by which synergistic effect of increased BCAAs levels and MS are thought to promote CV risk. One is that BCAAs were found to be a predictor of diabetes in general population [34, 35]. A new longitudinal data in a population-based cohort of nondiabetic British European and South Asian men, followed up for 19 years, concluded that BCAAs and AAAs may be focuses for identifying novel etiological mechanisms and potential treatment targets for diabetes in South Asian populations as they may contribute to their excess risk of diabetes [26]. Another is that BCAAs are related to other MS components such as elevated WC, elevated TG, and decreased HDL-C in animal and clinical study [24, 36]. A recent longitudinal study from Japan showed that plasma-free amino acids profile can predict the four-year risk for developing lifestyle-related diseases, including diabetes, dyslipidemia, and hypertension in a general Japanese population [37]. In our study, BCAAs were significantly associated with MS components (elevated WC, elevated TG, and decreased HDL-C) and MS. Accordingly it was inferred that BCAAs have dual role in CV risk by direct impairment of the heart via activation of the mTOR signaling pathway [6] or by indirect effects through the interaction between BCAAs and MS.

Our study had some limitations. Firstly, CV risk was evaluated by Framingham score rather than accurate coronary angiography. Secondly, a cross-sectional study cannot infer the causality between BCAAs, MS, and subsequent CVD. Therefore, more longitudinal studies are needed to investigate whether BCAAs levels associated with MS represent a new risk pattern identified CVD risk and accidental death in Asian population. Thirdly, some factors such as management of diabetes and hypertension, protein intakes, and exercises were not adjusted in our models which probably affected the results.

## 5. Conclusions

In conclusion, our results suggest that BCAAs not only appear to be an independent marker of adverse CV risk, but also exert synergistic effect with MS contributing to CV risk in middle-aged and elderly Chinese populations. From a practical standpoint, physicians should be aware that patients with concomitant MS and increased BCAAs levels are at increased risk of developing CVD. Intervention studies are needed to investigate whether a strategy to reduce BCAAs can impact on endpoints in patients with high CV risk.

## Supplementary Material

Supplementary Table 1 showed that BCAA was also the independent risk factor of MS.
Supplementary Table 2 showed that BCAA was also the independent risk factor of MS no matter male or female.
Supplementary Table 3 showed that BCAA was also the independent risk factor of high Framingham risk score no matter male or female.

## Figures and Tables

**Table 1:** Characteristics of the study participants according to BCAAs quartiles (*n* = 1302).

Characteristics	BCAAs (*μ*g/mL)					
	I	II	III	IV	*P* value	
	(<62.06, *n* = 325)	(62.06–70.01, *n* = 326)	(70.02–79.57, *n* = 325)	(≥79.57, *n* = 326)	Unadjusted	Adjusted by age and gender
Male, *n* (%)	20.9	34.1	38.2	46.9	<0.001	—
Age (years)	57.88 ± 6.14	59.06 ± 6.09	59.93 ± 5.67	59.05 ± 5.98	0.001	—
Current smoking, *n* (%)	18.8	17.6	18.2	17.22	0.054	—
Alcohol use, *n* (%)	16.9	16.1	17.5	18.42	0.764	—
Diabetes, *n* (%)	9.2	12.31	17.21	26.41	<0.001	<0.001
History of hypertension, *n* (%)	28.9	34.41	35.12	36.2	<0.001	—
Administration of ACEI or ARBs, *n* (%)	6.52	5.81	6.11	7.02	0.234	—
WC (cm)	80.67 (67.78, 92.5)	83.05 (69.87, 98.5)	84.77 (69.99, 102.20)	85.71 (69.20, 105.66)	<0.001	<0.001
BMI (kg/m^2^)	23.88 ± 3.05	24.26 ± 3.09	25.04 ± 3.11	24.99 ± 2.99	<0.001	<0.001
SBP (mmHg)	139.86 ± 20.54	139.44 ± 18.04	140.27 ± 20.81	139.98 ± 19.83	0.987	—
DBP (mmHg)	84.55 ± 13.10	83.70 ± 14.62	83.89 ± 15.51	83.62 ± 13.57	0.875	—
FPG (mg/dL)	5.26 (3.52, 17.26)	5.35 (4.01, 15.23)	5.49 (4.02, 19.77)	5.76 (3.46, 18.27)	<0.001	<0.001
HbA1c (%)	5.90 (4.20, 12.50)	5.90 (4.61, 13.11)	5.90 (4.30, 11.80)	6.00 (4.90, 12.51)	<0.001	<0.001
TC (mmol/L)	5.26 ± 0.93	5.13 ± 0.83	5.10 ± 0.83	5.22 ± 0.86	0.15	—
TG (mmol/L)	1.49 (0.50, 7.44)	1.70 (0.63, 6.40)	1.77 (0.58, 19.61)	2.00 (0.72, 16.42)	<0.001	<0.001
HDL-C (mmol/L)	1.43 (0.69, 4.78)	1.29 (0.71, 5.20)	1.26 (0.72, 5.19)	1.21 (0.66, 6.04)	<0.001	<0.001
LDL-C (mmol/L)	2.79 ± 0.76	2.70 ± 0.66	2.72 ± 0.69	2.77 ± 0.70	0.301	—
UA (mg/dL)	258.23 ± 68.76	281.40 ± 74.05	284.58 ± 81.56	303.00 ± 83.90	<0.001	—
Serum creatinine (mg/dL)	0.73 (0.43, 1.23)	0.76 (0.47, 1.24)	0.77 (0.49, 1.22)	0.82 (0.43, 1.25)	<0.001	—
eGFR (mL/min/1.73 m^2^)	96.88 ± 21.32	94.95 ± 16.50	95.09 ± 19.38	94.79 ± 20.34	0.334	—
BCAAs (*μ*g/mL)	56.91 ± 8.57	66.45 ± 9.52	73.89 ± 8.97	87.06 ± 9.62	<0.001	<0.001
L-Leu (*μ*g/mL)	20.54 ± 2.57	24.42 ± 2.07	27.60 ± 2.36	33.45 ± 4.13	<0.001	<0.001
L-Ile (*μ*g/mL)	10.22 (5.63, 14.42)	11.97 (8.66, 25.07)	13.54 (9.39, 18.84)	16.42 (11.66, 24.23)	<0.001	<0.001
L-Val (*μ*g/mL)	25.02 (11.04, 33.84)	30.20 (22.49, 37.50)	33.13 (25.78, 44.36)	38.48 (28.34, 56.66)	<0.001	<0.001
MS components 0, 1, 2, 3, 4, and 5 (%)	19, 29, 25, 16, 9, and 1	13, 28, 24, 23, 11, and 2	8, 22, 29, 23, 15, and 3	7, 17, 20, 30, 20, and 6	<0.001	<0.001
CVD risk score (%)	10.00 (1.50, 30.00)	13.71 (2.00, 30.00)	15.90 (1.70, 30.00)	18.4 (2.00, 30.00)	<0.001	<0.001
High Framingham CV risk score, *n* (%)	18.50	34.20	38.82	44.44	<0.001	<0.001

*Notes*. Data were presented as means (±SD) or median with range (minimum, maximum) as appropriate, and categorical variables are expressed as number (%). Means and proportions were compared by ANOVA, Fisher's exact test, and the chi-square test, respectively. *P* values testing the overall difference among BCAAs quartiles. BCAAs: branched-chain amino acids; ACEI: angiotensin converting enzyme inhibitor; ARBs: angiotensin receptor blockers; MS: metabolic system; CVD: cardiovascular disease; WC: waist circumference; BMI: body mass index; SBP: systolic blood pressure; DBP: diastolic blood pressure; HDL-C: high density lipoprotein cholesterol; LDL-C: low density lipoprotein cholesterol; FPG: fasting plasma glucose; HbA1c: hemoglobin A1c; TG: triglycerides; TC: total cholesterol; UA: uric acid; e-GFR: estimated glomerular filtration rate; L-Leu: L-leucine; L-Ile: L-isoleucine; and L-Val: L-valine. Definitions of hypertension, diabetes, and MS were in Materials and Methods. International system of units (SI) conversion: plasma glucose 1 mg/dL = 1/18 mmol/L; SUA 1 mg/dL = 59.5 mmol/L; and serum creatinine 1 mg/dL = 88.41 *μ*mol/L.

**Table 2:** Correlations between BCAAs and cardiometabolic risk factors.

Variables	BCAAs	L-Leu	L-Ile	L-Val
Age	0.083	0.079	0.070	0.066
Ln (waist circumference)	0.219^*∗*^	0.215^*∗*^	0.212^*∗*^	0.176^*∗*^
BMI	0.169^*∗*^	0.148^*∗*^	0.145^*∗*^	0.160^*∗*^
SBP	−0.002	−0.014	−0.012	0.02
DBP	−0.032	−0.033	−0.025	−0.021
Ln (FPG)	0.260^*∗*^	0.215^*∗*^	0.216^*∗*^	0.249^*∗*^
HbA1c	0.164^*∗*^	0.125^*∗*^	0.139^*∗*^	0.181^*∗*^
TC	0.005	0.007	0.04	0.032
Ln (TG)	0.264^*∗*^	0.234^*∗*^	0.230^*∗*^	0.240^*∗*^
Ln (HDL-C)	−0.268^*∗*^	−0.255^*∗*^	−0.281^*∗*^	−0.206^*∗*^
LDL-C	0.017	0.050	0.032	−0.016
UA	0.213^*∗*^	0.217^*∗*^	0.221^*∗*^	0.154^*∗*^
eGFR	−0.062	−0.089	−0.079	0.033
MS components	0.223^*∗*^	0.194^*∗*^	0.203^*∗*^	0.197^*∗*^
CVD risk score	0.204^*∗*^	0.198^*∗*^	0.240^*∗*^	0.144^*∗*^

*Notes*. ^*∗*^ *P* < 0.01. Data that were not normally distributed were Napierian logarithmically transformed before analysis. BCAAs: branched-chain amino acids; MS: metabolic system; CVD: cardiovascular disease; BMI: body mass index; SBP: systolic blood pressure; DBP: diastolic blood pressure; HDL-C: high density lipoprotein cholesterol; LDL-C: low density lipoprotein cholesterol; FPG: fasting plasma glucose; HbA1c: hemoglobin A1c; TG: triglycerides; TC: total cholesterol; UA: uric acid; e-GFR: estimated glomerular filtration rate; L-Leu: L-leucine; L-Ile: L-isoleucine; and L-Val: L-valine.

**Table 3:** Multiple logistic regression analyses of the relationship between BCAAs and MS.

Independent variable	Model 1		Model 2	
	OR (95% CI)	*P* value	OR (95% CI)	*P* value
BCAAs quartiles				
I	1		1	
II	1.56 (1.12–2.18)	<0.001	1.46 (0.98–2.23)	0.543
III	1.85 (1.32–2.58)	<0.001	1.78 (0.784–1.83)	0.405
IV	3.59 (2.59–5.0)	0.013	2.17 (1.41–3.34)	<0.001
L-Leu (*μ*g/mL)	1.09 (1.06–1.11)	<0.001	1.04 (1.02–1.07)	0.002
L-Ile (*μ*g/mL)	1.18 (1.13–1.23)	<0.001	1.08 (1.03–1.14)	0.002
L-Val (*μ*g/mL)	1.07 (1.05–1.09)	<0.001	1.02 (1.01–1.04)	0.011

*Notes*. CI: confidence interval; OR: odds ratio; BCAAs: branched-chain amino acids; MS: metabolic syndrome; L-Leu: L-leucine; L-Ile: L-isoleucine; and L-Val: L-valine. Definitions of BCAAs quartiles were in Statistical Analysis. Model 1: adjusting for age and gender; Model 2: adjusting for age, gender, current smoking status and drinking, and the administration of angiotensin converting enzyme inhibitors or angiotensin receptor blockers and MS components (systolic blood pressure, diastolic blood pressure, waist circumference, triglycerides and high density lipoprotein cholesterol, and fasting plasma glucose).

**Table 4:** Multiple logistic regression analyses of the relationship between BCAAs and high Framingham cardiovascular risk.

Independent variable	Model 1		Model 2		Model 3	
	OR (95% CI)	*P* value	OR (95% CI)	*P* value	OR (95% CI)	*P* value
BCAAs (*μ*g/mL)	1.03 (1.01–1.04)	<0.001	1.03 (1.02–1.04)	0.001	1.03 (1.00–1.04)	0.001
L-Leu (*μ*g/mL)	1.07 (1.03–1.11)	<0.001	1.06 (1.02–1.10)	0.001	1.06 (1.02–1.09)	0.029
L-Ile (*μ*g/mL)	1.18 (1.10–1.23)	<0.001	1.17 (1.11–1.22)	<0.001	1.16 (1.10–1.22)	0.008
L-Val (*μ*g/mL)	1.05 (1.03–1.07)	<0.001	1.05 (1.03–1.07)	<0.001	1.03 (1.01–1.06)	0.033

*Notes*. CI: confidence interval; OR: odds ratio; BCAAs: branched-chain amino acids; AAAs: aromatic amino acids; MS: metabolic system; L-Leu: L-leucine; L-Ile: L-isoleucine; and L-Val: L-valine. Model 1: unadjusting; Model 2: adjusting for MS and eGFR; and Model 3: adjusting for systolic blood pressure, diastolic blood pressure, fasting plasma glucose, triglycerides, high density lipoprotein cholesterol, uric acid, body mass index, estimated glomerular filtration rate, current drinking and smoking status, and administration of angiotensin converting enzyme inhibitors or angiotensin receptor blocker.

**Table 5:** Multiple logistic regression analyses of the relationship between BCAAs quartiles without or with MS and high Framingham cardiovascular risk.

Independent variable	Model 1		Model 2		Model 3	
	OR (95% CI)	*P* value	OR (95% CI)	*P* value	OR (95% CI)	*P* value
BCAAs quartiles without MS						
I	1		1		1	
II	1.68 (1.07–2.65)	0.019	1.67 (1.05–2.64)	0.029	1.88 (1.14–3.06)	0.013
III	2.13 (1.35–3.34)	0.001	2.14 (1.35–3.37)	0.001	2.01 (1.22–3.29)	0.005
IV	2.95 (1.83–4.73)	<0.001	2.97 (1.84–4.77)	<0.001	2.60 (1.54–4.38)	<0.001
BCAAs quartiles with MS						
I	1.39 (0.76–2.56)	0.291	1.26 (0.682–2.35)	0.457	1.57 (0.64–3.83)	0.540
II	4.40 (2.69–7.20)	<0.001	4.33 (2.63–7.13)	<0.001	2.40 (1.41–4.07)	<0.001
III	4.79 (2.96–7.73)	<0.001	4.64 (2.85–7.54)	<0.001	2.97 (1.70–5.18)	<0.001
IV	4.89 (2.93–7.89)	<0.001	5.18 (3.23–8.12)	<0.001	3.20 (1.83–5.61)	<0.001

*Notes*. CI: confidence interval; OR: odds ratio; BCAAs: branched-chain amino acids; MS: metabolic system; L-Leu: L-leucine; L-Ile: L-isoleucine; and L-Val: L-valine. Definitions of BCAAs quartiles were in Statistical Analysis. Model 1: unadjusting; Model 2: adjusting eGFR; and Model 3: adjusting for systolic blood pressure, diastolic blood pressure, fasting plasma glucose, triglycerides, high density lipoprotein cholesterol, uric acid, body mass index, estimated glomerular filtration rate, current drinking and smoking status, and administration of angiotensin converting enzyme inhibitors or angiotensin receptor blocker.
