# Supplementary material for: Relationship between Branched-Chain Amino Acids, Metabolic Syndrome, and Cardiovascular Risk Profile in a Chinese Population: A Cross-Sectional Study
Source: Int J Endocrinol. 2016 Jul 26;2016:8173905. doi: 10.1155/2016/8173905 (PMC4977397; doi:10.1155/2016/8173905)
Supplement: Supplementary file 1 — Supplementary Table 1 showed that BCAA was also the independent risk factor of MS. Supplementary Table 2 showed that BCAA was also the independent risk factor of MS no matter male or female. Supplementary Table 3 showed that BCAA was also the independent risk factor of high Framingham risk score no matter male or female. [file 8173905.f1.docx]

**Supplementary Table 1.** Multiple logistic regression analyses of the relationship between BCAAs and MS

| Independent variable | Model 1 | | Model 2 | |
| --- | --- | --- | --- | --- |
|  | OR (95% CI) | P-value | OR (95% CI) | P-value |
| BCAAs | 1.039 (1.029-1.048) | <0.001 | 1.012 (1.001-1.025) | 0.034 |

**Supplementary Table 2.** Multiple logistic regression analyses of the relationship between BCAAs and MS across to gender

| Independent variable | Male | | Female | |
| --- | --- | --- | --- | --- |
|  | OR (95% CI) | P-value | OR (95% CI) | P-value |
| BCAAs | 1.01 (1.00-1.02) | 0.041 | 1.01 (1.001-1.03) | 0.033 |

Notes: adjusting for age, gender, current smoking status and drinking, the administration of angiotensin converting enzyme inhibitors or angiotensin receptor blockers and MS components (systolic blood pressure, diastolic blood pressure, waist circumference, triglycerides and high density lipoprotein cholesterol; fasting plasma glucose).

**Supplementary Table 3.** Multiple logistic regression analyses of the relationship between BCAAs and high Framingham cardiovascular risk across to gender

| Independent variable | Male | | Female | |
| --- | --- | --- | --- | --- |
|  | OR (95% CI) | P-value | OR (95% CI) | P-value |
| BCAAs | 1.02(1.00-1.03) | 0.034 | 1.02(1.001-1.04) | 0.023 |

Notes: adjusting for systolic blood pressure, diastolic blood pressure, fasting plasma glucose, triglycerides, high density lipoprotein cholesterol, uric acid, body mass index, estimated glomerular filtration rate, current drinking and smoking status and administration of angiotensin converting enzyme inhibitors or angiotensin receptor blocker.
